# Quantification of Internalized Silica Nanoparticles via STED Microscopy

**DOI:** 10.1155/2015/961208

**Published:** 2015-06-01

**Authors:** Henrike Peuschel, Thomas Ruckelshausen, Christian Cavelius, Annette Kraegeloh

**Affiliations:** Leibniz-Institute for New Materials (INM), Campus D2 2, 66123 Saarbruecken, Germany

## Abstract

The development of safe engineered nanoparticles (NPs) requires a detailed understanding of their interaction mechanisms on a cellular level. Therefore, quantification of NP internalization is crucial to predict the potential impact of intracellular NP doses, providing essential information for risk assessment as well as for drug delivery applications. In this study, the internalization of 25 nm and 85 nm silica nanoparticles (SNPs) in alveolar type II cells (A549) was quantified by application of super-resolution STED (stimulated emission depletion) microscopy. Cells were exposed to equal particle number concentrations (9.2 × 10^10^ particles mL^−1^) of each particle size and the sedimentation of particles during exposure was taken into account. Microscopy images revealed that particles of both sizes entered the cells after 5 h incubation in serum supplemented and serum-free medium. According to the *in vitro* sedimentation, diffusion, and dosimetry (ISDD) model 20–27% of the particles sedimented. In comparison, 10^2^-10^3^ NPs per cell were detected intracellularly serum-containing medium. Furthermore, in the presence of serum, no cytotoxicity was induced by the SNPs. In serum-free medium, large agglomerates of both particle sizes covered the cells whereas only high concentrations (≥ 3.8 × 10^12^ particles mL^−1^) of the smaller particles induced cytotoxicity.

## 1. Introduction

Engineered nanomaterials (ENMs) are already out on the market and are found in a broad range of applications, ranging from everyday goods to electronics and biomedicine [1]. Examples are filler materials [2], cosmetics [3], and food products [4] as well as polishing agents, capacitors [1], and contrast agents [5]. Beyond that, many ENMs show promise for their use in novel and future applications [6]. In particular, in the biomedical field new ENMs are developed for targeted drug delivery, therapeutic, and theranostic purposes [7]. Considering the numerous fields of application as well as the broad variety of ENM types, it is critical to identify the relevant NP properties contributing to adverse health and environmental effects in order to design safe ENMs. Biologically relevant NP properties are considered to be intrinsic particle properties that might be modulated by interactions with molecules present in the environment.

To understand the mechanisms of NP cell interactions, it is important to identify and quantify NP targets within the body, including the location of internalized NPs on a cellular and subcellular level. NPs seem to enter cells via endocytotic as well as nonendocytotic pathways depending on size, surface chemistry, and shape [8–10]. In addition to physicochemical properties, it was demonstrated that internalization efficiency and particle induced toxicity* in vitro* depend on the presence of proteins in biological fluids [11]. In a recent study, the influence of the protein corona, associated with particles after dispersion in serum-containing media or biological fluids, on particle uptake has been attributed to a modulation of particle adhesion to the cellular surface [10, 12].

The mechanisms of toxicity induced by ENMs on a molecular level are still under investigation and due to a lack in standardization and comparability,* in vitro* studies apparently produce contradicting results regarding cytotoxic or even more subtle toxic effects. Besides differences in the particle properties, the protein corona, which modulates the cellular response to particles, may also give rise to these inconsistent results [13]. SNPs, for instance, are shown to induce reactive oxygen species (ROS) and DNA as well as chromosomal damage in various cell types [14–16]. Other studies demonstrate that SNPs enter cells but induce no cytotoxic or genotoxic effects [17, 18]. Thus, in order to elucidate how NPs induce specific cellular responses, it is critical to have information not only about administered (the initially added dose) and delivered (particles reaching the cell monolayer via diffusion and sedimentation) doses [19], but also about the cellular (particles associated with the cells) and intracellular doses (particles internalized by the cells). Ultimately, such data is also critical for a proper hazard assessment. For example, Geiser and Kreyling estimated that 1.4 × 10^11^ NPs per day are deposited in the lung, after exposition of a healthy individual to a moderate concentration of 3 × 10^4^ ambient particles cm^−3^ air volume. Even at the highest possible aerosol number concentration, an alveolar cell will maximally receive on average 120 NPs per hour [20]. Usually, during* in vitro* experiments, submerged cells are exposed to NPs dispersed in media. The diffusion, sedimentation, and agglomeration of these NPs, which are dependent on particle size and surface properties as well as density of the surrounding fluid, then have an impact on the delivered dose [21]. Therefore, Hinderliter et al. proposed a computational model to evaluate the fraction of the delivered dose that is deposited in an* in vitro* experiment [22].

Different approaches have been utilized to experimentally quantify the cellular or intracellular dose of NPs, such as atomic emission spectrometry [23], laser ablation ICP-MS [24], flow cytometry, imaging flow cytometry, and electron microscopy [25–28]. Of these, only electron microscopy enables direct counting of NPs [29], with the disadvantage of elaborate sample preparation. To quantify NP uptake, flow cytometry is used as a high throughput method. In contrast to this approach, confocal laser scanning microscopy (CLSM) retrieves information about the intracellular NP location. For example, CLSM has been employed to observe fluorescently labeled SNPs in cytosolic vesicles [30, 31]. As per the definition [32], the size range of NPs (1–100 nm) is below the classical optical resolution limit. Therefore, a promising method to study NP cell interactions is super-resolution fluorescence microscopy. Since the early nineties, several techniques have been developed to enhance resolution in optical microscopy. These techniques include (1) methods like 4Pi and I^5^M [33, 34], (2) single-molecule localization techniques, (3) structured illumination microscopy (SIM) [35–37], and (4) STED (stimulated emission depletion) microscopy [38]. In the first approaches, dual-beam interference between two opposing objectives is used for axial resolution improvement. Single-molecule localization techniques are based on blinking/switching of fluorophores and include PALM (photoactivated localization microscopy) [39], STORM (stochastic optical reconstruction microscopy) [40], and related methods [41–46]. In SIM, high frequency information of the specimen is transferred to a transmittable lower frequency range, whereas STED microscopy depletes fluorophores in the outer area of the point-spread function to sharpen the focus and, therefore, increases resolution in the lateral and/or axial plane. Using STED microscopy, it was demonstrated that 30 nm fluorescently labeled SNPs penetrated the nucleus of Caco-2 cells [47]. Using spinning disc and 4Pi confocal microscopy [33], Jiang et al. assessed the uptake of 8 nm D-penicillamine-coated quantum dots in live HeLa cells. The authors observed a dose-dependent increase in intracellular, as well as membrane-associated, fluorescence per cell area [48]. Although recently an approach has been described to quantify internalized 1000, 400, and 250 nm sized polystyrene particles using CLSM in combination with high-throughput FACS data [49], the quantification of absolute numbers of NPs is still a challenge. Optical imaging techniques with enhanced resolution are needed to accurately determine the intracellular delivery of particles smaller than 200 nm in diameter.

In the present study, we used for the first time STED microscopy image stacks to quantify internalized fluorescently labeled 25 nm and 85 nm sized SNPs in A549 cells, as a model for lung epithelial cells. Image segmentation was applied to differentiate between internalized and attached NPs within 3D image stacks of whole cells. Here, the quantification of internalized SNPs is not based on measuring the mean fluorescence intensity of single particles or of cell-associated particles like in other approaches, but on segmented objects. Cells were exposed to identical initial particle concentrations (administered dose) and the intracellular doses were determined. In order to evaluate the role of proteins in NP cell interactions [11, 12], cells were exposed to SNPs in the presence and absence of serum proteins. Furthermore, the cytotoxicity of these SNPs was investigated with regard to the protein content in the surrounding medium.

## 2. Material and Methods

### 2.1. NP Synthesis and Labeling

All chemicals used for particle synthesis were supplied from Sigma-Aldrich (Taufkirchen, Germany) in the highest purity available. Atto647-NHS ester was purchased from Atto-Tec (Siegen, Germany). All procedures, which involved the active Atto-NHS ester, were performed under exclusion of moisture and light.

SNPs were synthesized as described before [47]. After synthesis, the particles were purified by dialysis against MilliQ water followed by filtration through a sterile 0.2 *μ*m membrane. To confirm the complete dye incorporation into the SNPs, a 100 *μ*L aliquot was centrifuged through a 30 kDa membrane followed by fluorescence spectroscopy (Spex FluoroMax-3, Horiba Scientific GmbH, Germany).

### 2.2. Particle Characterization

A series of TEM micrographs of dried nanoparticle dispersions obtained by electron microscopy (Philips CM200 FEG, FEI Company, Netherlands) was selected to estimate the average primary particle size. Samples were prepared by immersion of a 200-mesh carbon-coated copper grid into the nanoparticle suspension. ImageJ software from the National Institutes of Health (http://rsb.info.nih.gov/ij/) was used to estimate the mean particle size and particle size distribution.

Dynamic light scattering (DLS, Dyna Pro Titan, Wyatt Technology Europe GmbH and Nanotrac-Ultra, Microtrac Europe GmbH, Germany) was used to estimate the average hydrodynamic diameter of NPs dispersed in water and in cell culture medium. DLS measurements of 25 nm particles dispersed in serum-containing medium were not possible because protein and particle signals overlapped.

The zeta potential was measured with a Nanosizer Z (Malvern Instruments, Worcestershire, UK) in water at 150 V using 10^−3^ M KCl as background electrolyte. Each sample underwent three series of measurements (with each series comprising 40 measurements). In cell culture medium the zeta potential was measured at 20 V. Analytical centrifugation was performed with a LUMiSizer Dispersion Analyzer (LUM GmbH, Germany) by software controlled centrifugation of 1.4 mL of the sample dispersion. Size distribution and histograms were calculated using proprietary SEP View 6 software.

The specific surface area of the SNPs used in this study was calculated by comparison with unlabeled SNPs prepared by the same protocol. Briefly, the specific surface area of bare SNPs with a size of 23, 35, and 72 nm was determined by BET analysis via nitrogen adsorption (Autosorb-6B, Quantachrome Instruments, USA). A simple linear regression model (*y* = 143.2 − 1.505*x*, with *y* = surface area [m^2^/g], *x* = particle diameter [nm], and *r* ^2^ = 0.999) was used to extrapolate the BET surface area for Atto647N labeled particles assuming no significant difference in the adsorption behavior and porosity of bare and labeled silica. Nanoparticle number concentrations were calculated based on the EM derived particle diameter and the SiO_2_ content, which was obtained by ICP-OES (Ultima 2, Horiba JobinYvon, Japan). Endotoxin tests were performed using the LAL Gel Clot Assay (Lonza) according to Kucki 2012 [50].

### 2.3. Cell Culture

The cell line A549 as model for human alveolar epithelial type II cells was obtained from the German Collection of Microorganisms and Cell Culture (DSMZ, Braunschweig, Germany) and was maintained in a humidified incubator (37°C, 9% CO_2_, and pH 7.4) in Dulbecco's modified Eagle medium (DMEM, Gibco, Life Technologies, USA) supplemented with 10% fetal bovine serum (FBS, PAN biotech, Germany). After reaching ~80% confluence, cells were dislodged by using 0.05% trypsin containing 0.02% EDTA. For the analysis of particle uptake, A549-pAcGFP1-Mem cells were used as described in Schumann et al. 2012 [30]. These cells express a green fluorescent protein derivative fused to the* N*-terminal membrane targeting signal of neuromodulin for membrane labeling (Clontech, Mountain View, CA). A549-pAcGFP1-Mem cells were maintained in selective cell culture medium (DMEM with 10% FBS) containing 200 *μ*g mL^−1^ G418.

### 2.4. Exposure of Cells to NPs

For confocal imaging, A549-pAcGFP1-Mem cells were seeded on glass coverslips at a density of 1 × 10^5^ cells mL^−1^ in 12-well plates (Greiner Bio-One, Frickenhausen, Germany) and allowed to attach for at least 20 h. Nanoparticle dispersions were freshly prepared in serum-free or serum-containing medium. Exposure time for all experiments was 5 hours, unless otherwise stated. Cells were incubated with Si-25-FD and Si-85-FD dispersions at a concentration of 9.2 × 10^10^ particles mL^−1^. For scanning electron microscopy, 1 × 10^5^ cells mL^−1^ were incubated on glass coverslips in 12-well plates with 1, 10, and 100 *μ*g mL^−1^ unlabeled SNPs in presence and absence of serum. For cytotoxicity experiments, A549 cells were seeded in 96-well plates at a density of 1 × 10^5^ cells mL^−1^. Here, particle dispersions of Si-25 and Si-85 at concentrations of 1, 10, 50, and 200 *μ*g mL^−1^ were used. Control samples were not exposed to NPs.

### 2.5. Immunostaining

After the incubation, cells were washed two times with DPBS, fixed with 4% paraformaldehyde in PBS for 30 min, and permeabilized with 0.2% Triton X-100 for 15 min at room temperature. Lamin-B (goat polyclonal antibody, sc-6216, Santa Cruz, Heidelberg, Germany) and secondary antibody Alexa Fluor 546 (donkey anti-goat, A11056, Invitrogen, Darmstadt, Germany) were used to stain the lamina of the nucleus. Cells were mounted on glass slides with Mowiol/DABCO (Sigma Aldrich, Taufkirchen, Germany).

### 2.6. STED and Confocal Microscopy

A confocal laser scanning microscope Leica TCS-SP5 STED (Leica Microsystems, Mannheim, Germany) with a Leica HCX PLAN APO 100x/1.4 oil immersion objective was used. Specimens were imaged using the 488 nm laser line of an Argon laser for excitation of the cellular membrane label (AcGFP1) and a 561 nm DPSS laser for excitation of the nuclear membrane label (Alexa Fluor 546). Cellular structures were imaged in confocal mode. The NPs, labeled with Atto647N, were imaged in STED mode (after incubation of cells in presence of serum) using a pulsed 635 nm laser diode (PicoQuant, Berlin, Germany) for excitation and an infrared laser (MaiTai, Spectra Physics, Santa Clara, United States) running at 750 nm for STED depletion. After incubation of cells in absence of serum, Atto647N labeled particles were imaged in confocal mode using the pulsed 635 nm laser diode for excitation. APD modules (Perkin-Elmer SPCM-AQRH) were used for detection of particle signals received in STED mode, whereas cellular structures and particle signals received in confocal mode were detected with the internal analog PMT detectors. The confocal pinhole was set to 1 AU to optimize z-sectioning. Images and z-stacks were recorded sequentially. A step size of 130 nm was chosen. The pixel size was set to 30 nm for STED and 60 nm for confocal images to avoid undersampling. To determine the 3D experimental point-spread function (PSF) of the system, 40 nm dark red fluorescent beads (F8789, Invitrogen) were imaged in STED mode, and 100 nm fluorescent multicolor beads (T7279, Invitrogen) were used for conventional confocal imaging. The full width at half maximum (FWHM) of the experimental PSF in the focal plane, as indicator for the lateral resolution, was 76 nm in STED mode and 277 nm in diffraction-limited confocal mode.

### 2.7. Image Processing

STED and confocal data were deconvolved using an iterative maximum likelihood algorithm implemented in Huygens Professional (SVI, Hilversum, Netherlands) and experimentally (for confocal mode) or theoretically (for STED mode) determined PSFs.

To discriminate between NPs inside and outside the cell the “Surface Renderer” tool of the Huygens software was used. The position of the isosurface was set at an intensity threshold calculated by the Otsu algorithm [51]. Thereby retrieved 3D region was set as region of interest (ROI). Remaining holes in the isosurface were closed with an open-close algorithm with a voxel number of two. To extend the ROI to the whole cell, the implemented “fill inner and cutoff cavities” algorithm was applied. If several cells were imaged in one frame, the segmented objects were additionally chosen by a manually drawn mask. In the NP channel, after the Otsu-thresholding, a watershed segmentation with a sigma of 2.0 *μ*m for the beforehand Gaussian filter was used to separate small NP agglomerates. Objects below an intensity value of 5% of the range between threshold and intensity maximum were discarded. All in this manner segmented objects of the NP channel inside the ROI of the cell were counted. The image processing workflow is illustrated in Figure 10.

### 2.8. Sedimentation, Diffusion, and Dosimetry Model (ISDD) Simulations

To investigate the effect of sedimentation and diffusion on the SNPs used in this study and to determine the delivered dose, the ISDD model, presented by Hinderliter et al. [22], was applied. The following parameters were applied: the SiO_2_ mass concentration was 1.2 *μ*g SiO_2_ mL^−1^ (25 nm SNPs) and 50 *μ*g SiO_2_ mL^−1^ (85 nm SNPs), corresponding to the used particle number concentration of 9.2 × 10^10^ mL^−1^. A density of 1.8 g cm^−3^ was assumed for the SNPs. The number of particles per agglomerate was set to 1 and additionally to 3 or 4 particles per cluster. Other input parameters were the height of the medium column above the cells (5 mm, measured), its volume (1 mL), the temperature (37°C), and the deposition time (5 h).

### 2.9. Scanning Electron Microscopy of A549 Cells

After incubation, cells were fixed with 2.5% glutaraldehyde for 30 min at room temperature and rapidly dehydrated in a graded ethanol series and hexamethyldisilazane (HMDS). Samples were gold-palladium coated for high vacuum mode imaging and analyzed with an ESEM Quanta 400 FEG (FEI Company, Hillsboro, USA) microscope.

### 2.10. Membrane Integrity (LDH Assay)

The activity of lactate dehydrogenase (LDH) in the cell culture medium as an indicator for cell membrane damage after treatment with SNPs was measured using the CytoTox-ONE Homogenous Membrane Integrity Assay kit (Promega) according to the manufacturers' instructions. Cells incubated with medium only were used as negative controls and cells treated with Triton-X 100 were used as positive controls. A no cell control was included to measure the background fluorescence of the culture medium. After particle exposure, medium (50 *μ*L) from each well was placed in a black 96-well plate and 50 *μ*L CytoTox-ONE reagent was added and incubated for 10 min in the dark. Fluorescence was measured at excitation and emission wavelengths of 560 nm and 590 nm with a Tecan Microplate reader (Molecular Devices). Interference of the used SNPs with the assay was excluded prior to analysis by measuring the fluorescence of Triton-X-100 lysed cells in presence of 200 *μ*g mL^−1^ SNPs. No changes in the fluorescence signals could be observed. At 590 nm the SNPs do not absorb light.

### 2.11. Statistics

Results are presented as means and standard deviation (SD). Statistical comparisons were made with unpaired Student's *t*-test at a 95% confidence level. Differences in the viability of A549 cells were considered significant at *p* < 0.05.

## 3. Results 

### 3.1. Physicochemical Properties of SNPs

In this study, cells were exposed to amorphous SNPs with diameters of 25 nm (Si-25) and 85 nm (Si-85). For microscopy analysis and subsequent quantification, the NPs were fluorescently labeled using Atto647N (Si-25-FD and Si-85-FD, resp.). The physicochemical properties of the particles are shown in Table 1. The mean particle diameters as determined from TEM micrographs were 25 ± 3 nm (Si-25), 24 ± 2 nm (Si-25-FD), 84 ± 7 nm (Si-85), and 85 ± 8 nm (Si-85-FD), respectively. The hydrodynamic diameter of each type of particle dispersed in water or serum-free medium (Dulbecco's modified Eagle medium (DMEM)) was found to be comparable to its corresponding mean particle diameter, determined by EM. The diameter of 25 nm particles dispersed in serum-containing medium (DMEM + 10% FBS) could not be measured due to significant interference signals of medium proteins. Therefore, concentration-dependent particle stability in serum-containing media was measured using 94 nm SNPs (see Additional file 1 in Supplementary Material available online at http://dx.doi.org/10.1155/2015/961208).

All particles exhibited a negative zeta potential in all of the three media measured (Table 1). However, in the presence of serum-free and complete culture medium a reduction of the absolute zeta potential values was observed. The specific surface area of 25 nm particles was calculated to be almost three times larger than the specific surface area of 85 nm particles. No endotoxin contamination was detected in dispersions of Si-25 and Si-85 particles.

### 3.2. Simulation of NP Sedimentation and Diffusion

In this study, cells were grown in a standard submersed cell culture system and then exposed to NPs by exchange of the medium with NP-containing medium. The initial concentration (administered dose) of Si-25-FD and Si-85-FD was 9.2 × 10^10^ particles mL^−1^, respectively. Since it is known that NPs reach the cell surface by sedimentation and diffusion, which depend on the particle size, the delivered dose was simulated by using the ISDD model for noninteracting spherical particles and their agglomerates presented by Hinderliter et al. [22]. The model is based on Stokes' law, which predicts particle sedimentation velocity, and the Stokes-Einstein equation, which describes the diffusion coefficient of the particles. The delivered dose in serum-containing medium was calculated assuming a cluster size of one, according to the DLS analysis (Table 1), and taking into account the stabilizing effects of serum [52]. The simulation revealed that after 5 h the number fraction of deposited particles was 26.5% and 20.4% for Si-25 and Si-85 particles, respectively (Table 2).

### 3.3. Uptake of SNPs in the Presence and Absence of Serum

**Figure 1:**
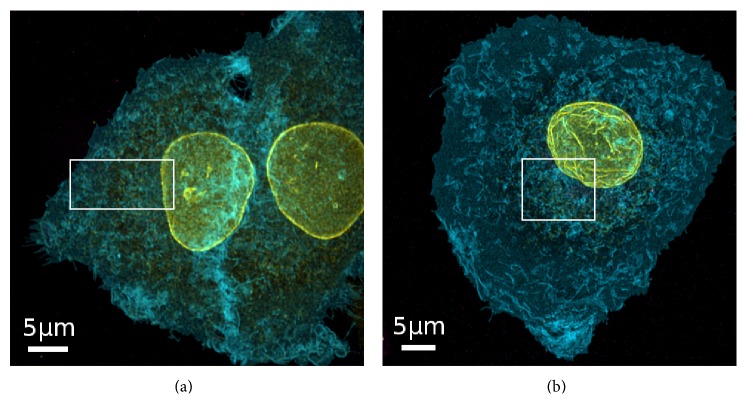
Overviews of A549 cells exposed to SNPs. Images are maximum intensity projections obtained by taking z-stacks of whole A549 cells that had been exposed to Si-25-FD (a) or Si-85-FD (b) particles at an initial number concentration of 9.2 × 10^10^ SNPs mL^−1^ for 5 h. For microscopy studies, A549-pAcGFP1-Mem cells were used, expressing a fluorescent protein derivative fused to the* N*-terminal membrane targeting signal of neuromodulin for membrane labeling (cyan). The nuclear lamina is shown in yellow. SNPs (magenta), labeled with Atto647N, were imaged in STED mode and the two other structures in conventional confocal mode. White boxes indicate the areas shown in more detail in Figures 2 and 3. Contrast and brightness were adjusted in all images for better illustration.

**Figure 2:**
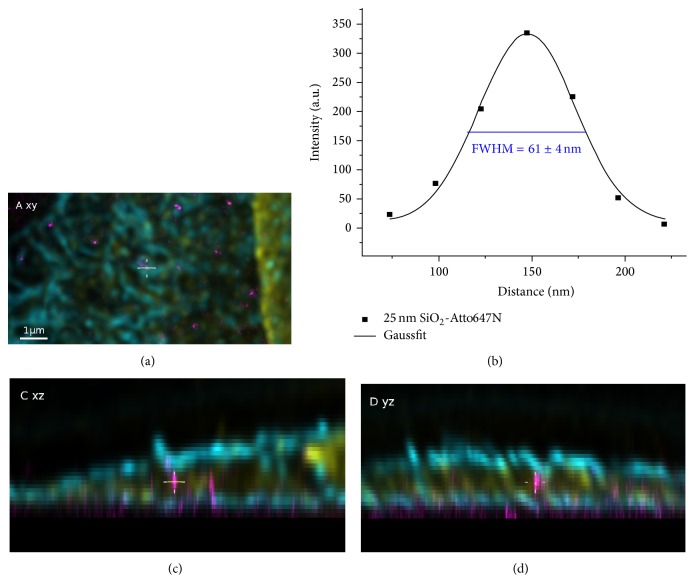
Enlarged sections of an A549 cell exposed to Si-25-FD particles for 5 h. ((a), (c), and (d)) They are orthogonal sections of the area indicated in Figure 1(a). Intersecting planes pass the middle of the image (cross hair). SNPs (magenta), membrane (cyan), and nuclear lamina (yellow). (b) An intensity plot through a sample nanoparticle (yellow line) within the cell. The full width of half maximum (FWHM) was 61 ± 4 nm, which is well below the classical optical resolution limit (error: standard deviation of Gaussian fit).

**Figure 3:**
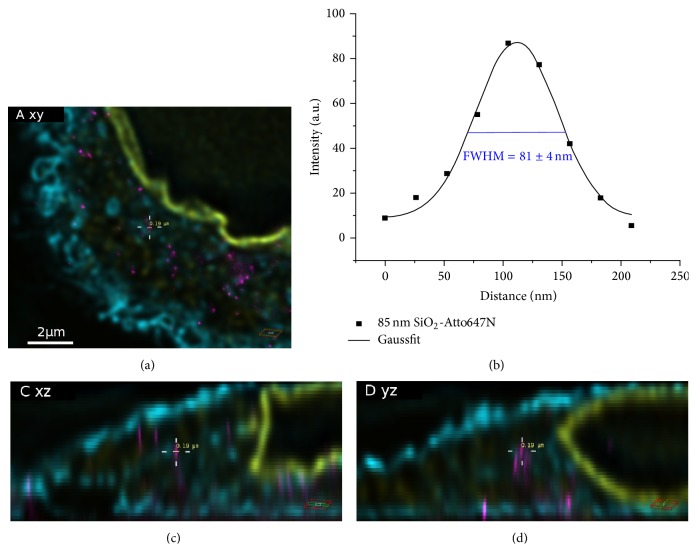
Enlarged sections of an A549 cell exposed to Si-85-FD particles for 5 h. ((a), (c), and (d)) They are orthogonal views of the area indicated in Figure 1(b). Intersecting planes pass the middle of the image (cross hair). SNPs (magenta), membrane (cyan), nuclear lamina (yellow). (b) is an intensity plot through an example nanoparticle (yellow line). The full width of half maximum (FWHM) was 81 ± 4 nm, indicating that single NPs can be detected in the cytosol (error: standard deviation of Gaussian fit).

In order to determine the uptake of fluorescently labeled SNPs, A549 cells expressing a green fluorescent protein targeted to the cytoplasmic membrane (A549-pAcGFP1-Mem) were exposed to 9.2 × 10^10^ SNPs mL^−1^ in complete or serum-free medium for 5 h. This exposure time was chosen because a significant amount of particles was expected to become internalized by that time [17]. In the two example overviews of cells exposed to particles in serum supplemented medium (Figure 1), only the cellular structures imaged in confocal mode can be seen. Nevertheless, the STED signals of the particles were recorded during image analysis. Enlarged sections of the cells depicted in Figure 1 are shown in Figures 2 and 3. In these sections, the NPs imaged in STED mode are clearly visible. The particles appeared to be distributed throughout the cells. No large particle agglomerates were detected inside or outside of the cells (Figures 2 and 3). A fluorescence intensity plot of a sample 25 nm particle (Figure 2, yellow line) within the cell revealed a full width of half maximum (FWHM) of 61 ± 4 nm (the error corresponds to the standard deviation of Gaussian fit). This value was lower than the mean FWHM of the point-spread function that had been obtained by measurements using fluorescently labeled (40 nm) latex beads (PSF_STED_ ≈ 76 nm). On the other hand, the FWHM determined by STED imaging was two-times larger than the particle diameter determined by TEM. In contrast, an intensity plot through an exemplary 85 nm particle (Figure 3, yellow line) resulted in a FWHM of 88 ± 4 nm (error: standard deviation of Gaussian fit), corresponding to the particle diameter determined by TEM. In both cases, particle size values measured by STED imaging were well below the classical optical resolution limit. Although STED imaging did not allow single 25 nm particles to be resolved, single 85 nm particles were clearly resolved.

**Figure 4:**
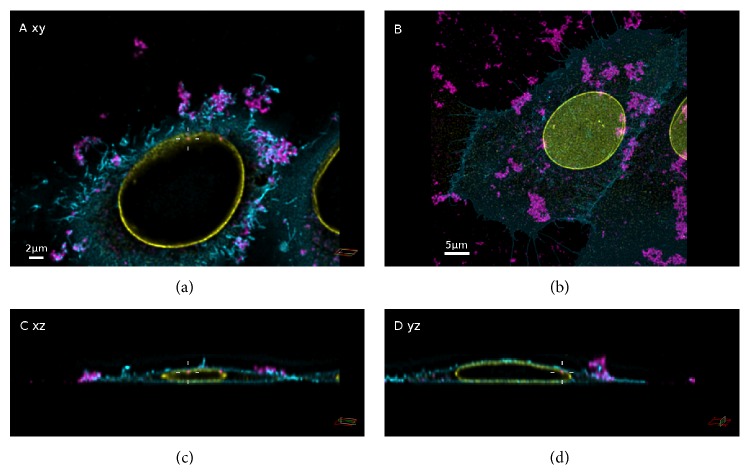
Confocal images of A549 cells exposed to Si-25-FD particles in the absence of serum. Maximum intensity projection (b) and orthogonal views (*xy*,* xz*, and* yz*) of one cell within the same section ((a), (c), and (d)). Intersecting planes pass the middle of the image (cross hair). SNPs (magenta), membrane (cyan), and nuclear lamina (yellow).

**Figure 5:**
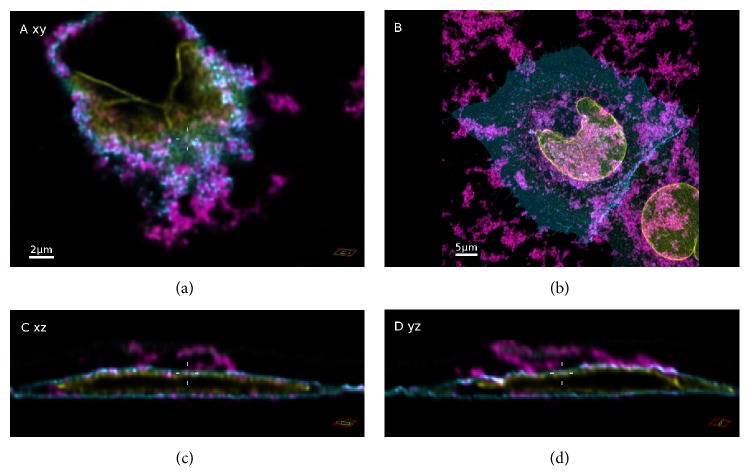
Confocal images of A549 cells exposed to Si-85-FD particles in the absence of serum. Maximum intensity projection (b) and orthogonal views (*xy*,* xz*, and* yz*) of one cell within that image section ((a), (c), and (d)). Intersecting planes pass the middle of the image (cross hair). SNPs (magenta), membrane (cyan), and nuclear lamina (yellow).

After exposition of A549 cells to either 25 nm or 85 nm SNPs in absence of serum, large (up to a few *μ*m in size), irregular particle agglomerates were observed (Figures 4 and 5). In contrast, DLS measurements did not indicate formation of agglomerates under these conditions (Table 1). The brightness of these agglomerates saturated the avalanche photodiode (APD) used for detection of the particle fluorescence signals in STED mode, resulting in the automatic power-down of the APD. Therefore, confocal microscopy had to be employed to acquire the image stacks instead. Orthogonal sections of confocal image stacks revealed that micrometer-sized NP agglomerates were only detected outside of the cells. These agglomerates appeared to be tightly attached to the cytoplasmic membrane. Under serum-free conditions the particles also exhibited a high tendency to attach to the surface of the coverslips. These attached particles were not removed by washing steps during sample preparation. Nevertheless, fluorescence signals were also detected inside the lumen of A549 cells, resembling either single particles or small particle agglomerates. Neither in presence nor in absence of serum were NP signals detected in the cell nucleus.

### 3.4. Quantification of SNP Internalization

The confocal images indicated that, in absence of serum, the SNPs tended to form agglomerates. As stated above, in confocal microscopy the fluorescence of the agglomerates saturated the APD detectors. Furthermore, a compensatory reduction of the intensity of the excitation laser caused the fluorescence of single NPs to drop below background. Therefore, only data from cells exposed to NPs in the presence of serum were used for quantification of particle internalization. Segmentation of the corresponding image data for quantification was performed as described in Material and Methods. Quantitative results of SNP internalization obtained by image processing are listed in Table 3. Approximately twenty cells were used to quantify the number of internalized particles at each particle size. After the A549 cells were exposed to either 25 nm particles or 85 nm particles, 117 ± 126 objects per cell or 338 ± 171 objects per cell were detected, respectively. The large deviations in the number of objects can be related to differences in cell size (Additional file 2). The measured difference in the number of objects per cell was found to be statistically significant (*p* < 0.001). In Table 3 the number of objects is also given per cell area. These values also indicate that a greater number of the larger particles were internalized by the cells. Nevertheless, after exposition to both particle sizes, the number of objects per cell was of the same order of magnitude. The mean intensity values of the segmented objects indicate that the objects corresponding to 85 nm particles exhibited a higher fluorescence intensity compared to the 25 nm particles. For 25 nm particles a maximum dye content of 11 molecules per particle was calculated, which is 600 times less than the maximum dye content of 6690 molecules per particle, calculated for 85 nm particles. Since these are theoretical calculations under the assumption of 100% coupling yield without considering quenching effects, a smaller difference of fluorescence intensity between the two particle sizes can be expected. Detailed information on microscopy data is given in Additional file 2.

### 3.5. Influence of Particle Exposure on Cell Morphology

**Figure 6:**
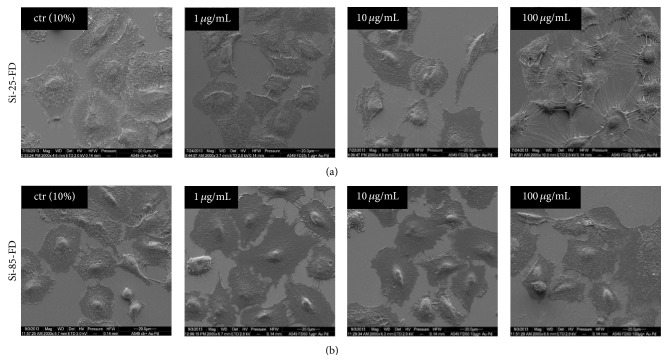
SEM micrographs of human type II alveolar epithelial cells (A549) incubated with Si-25-FD and Si-85-FD in serum-containing medium. No change in cell morphology was observed after exposure to 1, 10, or 100 *μ*g mL^−1^ SNPs for 5 h. Untreated cells serve as controls (ctr).

**Figure 7:**
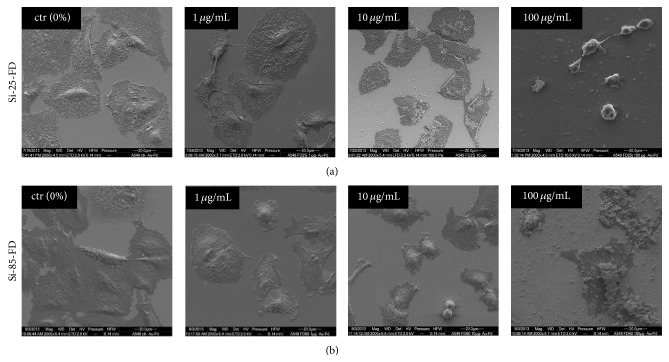
SEM micrographs of A549 cells incubated for 5 h with Si-25-FD and Si-85-FD in serum-free medium. SiO_2_ concentrations of 1 and 10 *μ*g mL^−1^ Si-25-FD induced no changes in cell morphology. After exposure to 100 *μ*g mL^−1^ Si-25-FD (a) A549 cells are round in cell shape compared to unexposed cells (ctr). At a concentration of 10 *μ*g mL^−1^ SiO_2_ and higher, both particle sizes formed agglomerates and bound to the cell membrane. Incubation with 1, 10, or 100 *μ*g mL^−1^ Si-85-FD particles (b) induced no changes in cell morphology.

Exposition of A549 cells to 9.2 × 10^10^ particles mL^−1^, corresponding to mass concentrations of 1.2 *μ*g SiO_2_ mL^−1^ (25 nm SNPs) and 50 *μ*g SiO_2_ mL^−1^ (85 nm SNPs), was not found to exert a detectable influence on cell morphology, regardless of the medium composition (Figures 1, 4, and 5). This finding was further analyzed by scanning electron microscopy (SEM), including lower and higher NP concentrations. SEM micrographs of cells exposed to either of both particle sizes in complete medium showed that cells underwent no change in cell morphology compared to untreated cells, regardless of the SiO_2_ concentrations employed (1 *μ*g mL^−1^, 10 *μ*g mL^−1^, and 100 *μ*g mL^−1^ SiO_2_) (Figures 6(a) and 6(b)). Similarly, after cells were exposed to 1 and 10 *μ*g SiO_2_ mL^−1^ in serum-free medium, SEM micrographs showed that cell morphology was not affected compared to untreated cells (Figures 7(a) and 7(b)). However, after exposing cells to 100 *μ*g mL^−1^ of Si-25-FD in serum-free medium, the cells appeared rounded. In contrast, after cell exposure to 100 *μ*g mL^−1^ of Si-85-FD under the same conditions, cell morphology did not change, although cells were highly decorated with particle agglomerates.

### 3.6. Particle Effects on Membrane Integrity

**Figure 8:**
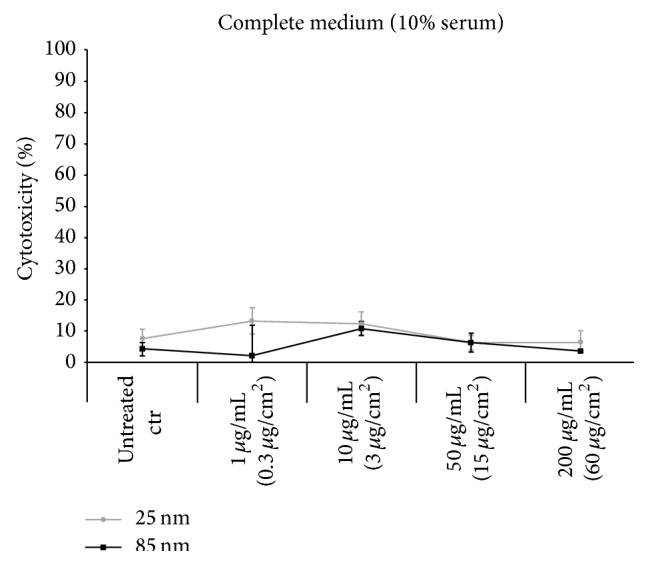
No membrane damage in the presence of serum proteins. A549 cells were incubated for 5 h with increasing concentrations (1, 10, 50, and 200 *μ*g mL^−1^) of Si-25 and Si-85 in complete medium (DMEM with 10% FBS). Concentrations in brackets refer to the area of a 12-well plate. LDH assay of supernatant was performed and no membrane damage could be observed compared to the unexposed control cells. Percent of cytotoxicity ± SD of 3 independent experiments is shown.

**Figure 9:**
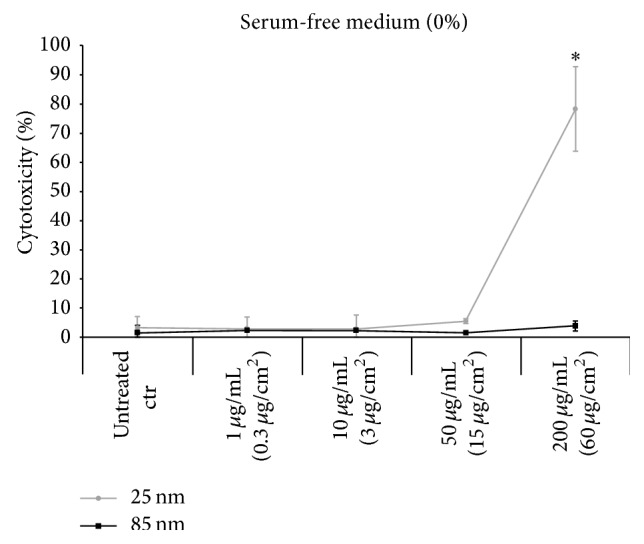
Membrane damage in the absence of serum proteins. A549 cells were incubated with increasing concentrations (1, 10, 50, and 200 *μ*g mL^−1^) of Si-25 and Si-85 in serum-free medium (DMEM with 0% FBS). LDH assay was performed after 5 h incubation. Only exposure to 200 *μ*g mL^−1^ Si-25 induced cell damage compared to unexposed control cells. Error bars represent SD of 3 independent experiments. ^*^Significantly different from untreated controls, *p* ≤ 0.05.

**Figure 10:**
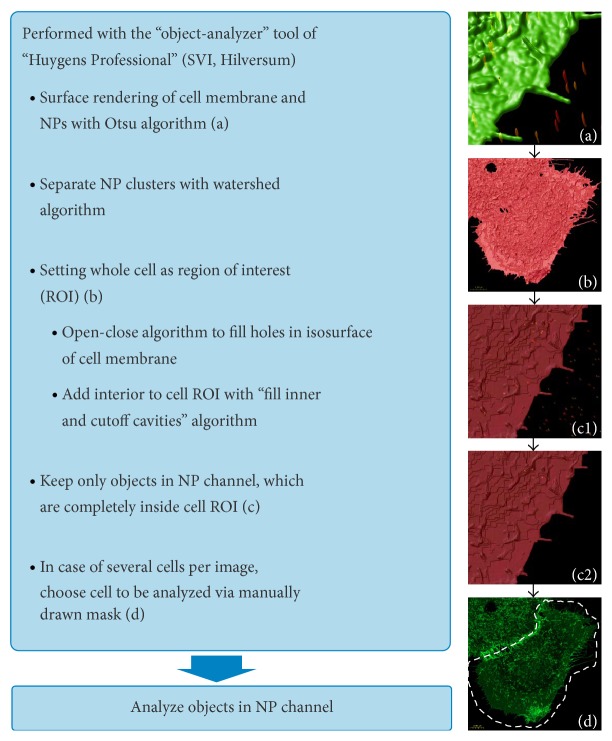
Image processing workflow.

Confocal images as well as SEM micrographs indicated that particles adhered to the cell membrane, especially in absence of serum. Therefore, the effect of SNPs on membrane integrity was analyzed using the lactate dehydrogenase (LDH) assay. After the incubation of A549 cells in presence of 1, 10, 50, or 200 *μ*g mL^−1^ of Si-25 or Si-85 particles in complete medium, no membrane damage was detected (Figure 8). In serum-free medium, only at the highest concentration of Si-25 particles (200 *μ*g mL^−1^), an increase in LDH activity was measured (Figure 9). In this case, about 90% of the cells exhibited LDH leakage. In contrast, membrane damage was not observed after incubation of cells in presence of 200 *μ*g mL^−1^ Si-85 particles dispersed in serum-free medium. Thus, at the particle concentration applied to microscopy analysis, no cytotoxicity was induced. Membrane damage was only observed in absence of serum at particle concentrations greater than 3.8 × 10^12^ 25 nm particles mL^−1^.

## 4. Discussion

Determining the NP dose cells receive during exposure to NPs* in vitro* and* in vivo *is essential in order to interpret biological responses with regard to assessing the risk of ENMs and evaluating drug delivery efficiency. Depending on the particle properties, various techniques have been employed to determine the cellular or even intracellular dose. In the present study, A549 cells were exposed to well-defined fluorescently labeled 25 and 85 nm amorphous SNPs in order to quantify internalized particles. NP quantification was achieved by processing 3D microscopy image stacks. Since the diffraction-limited resolution of CLSM is not sufficient to study the number of NPs inside cells [53], we imaged SNPs using STED microscopy. Furthermore, STED has been demonstrated to provide detailed information about the intracellular distribution and agglomeration state of 130 nm silica particles and 25 nm and 85 nm SNPs [17, 47]. In addition, the detection of individual silica particles by STED has been proven by a correlative STED SEM approach [17].

### 4.1. Particle Internalization Efficiency

After exposing A549 cells to 25 nm or 85 nm particles at 9.2 × 10^10^ SNPs mL^−1^ for 5 h, approximately 10^2^ objects were detected intracellularly. Analysis of the lateral object widths found within cells treated with 85 nm particles revealed that single 85 nm particles could be resolved by STED analysis (Additional file 3b). Additionally, 90% of the detected objects had a lateral object width of less than 150 nm and were considered to represent separated particles. Objects with a lateral width of more than 150 nm (10% in case of the 85 nm particles) were regarded as particle agglomerates. The largest agglomerate had a width of 578 nm. To estimate the total number of particles within intracellular agglomerates, one-, two-, and three-dimensional agglomerate models were applied, assuming packing densities of 0.91 (two-dimensional) and 0.74 (three-dimensional), respectively. Taking these models into account, the number of 85 nm SNPs per cell was estimated to range from 412 in case of one-dimensional agglomerates to 585 representing two-dimensional agglomerates to 957 in case of three-dimensional agglomerates (Additional file 4b).

In comparison, single 25 nm NPs could not be resolved by STED imaging as indicated by the measured FWHM value (61 nm) of a sample particle (Figure 2, Table 1). In case of the 25 nm particles, 58% of the detected objects had a lateral width of <75 nm, representing either single particles or small agglomerates. Depending on the agglomeration model applied, the number of 25 nm SNPs per cell was estimated to range from 404 in case of one-dimensional agglomerates to 1657 representing two-dimensional agglomerates to 7772 in case of three-dimensional agglomerates (Additional file 4a). Thus, taking intracellular particle agglomeration into account, it can be concluded that cells accumulated three- to eightfold higher numbers of the 25 nm particles compared with the 85 nm particles.

According to light scattering analysis and analytical centrifugation, the particles used in this study were shown to be well separated, when dispersed in either serum-containing medium or serum-free medium. In contrast, by microscopy large particle agglomerates were observed to cover the cells in the absence of serum.

In a TEM study, Rothen-Rutishauser et al. showed that, after exposing A549 cells to 9 × 10^11^ 15 nm polymer-coated gold NPs per milliliter for 1 h, 5365 NPs were internalized by the cells [53]. In comparison to the study described here, the cells were exposed to a significantly higher dose of NPs for a shorter time in absence of serum. After exposing A549 cells to citrate-coated gold NPs in presence of serum, 2600 and 3575 particles were quantified intracellularly after 1 h and 4 h, respectively [29]. In this case, a dose of 1 × 10^11^ particles mL^−1^ was administered. These values indicate that particle internalization is a time-dependent process, as expected when considering an active accumulation process. Similar trends have been found by Chithrani et al. [23] and Lesniak et al. [11] for the uptake of gold NPs and SNPs, respectively. In addition to time, the extracellular particle concentration is expected to influence uptake efficiency. For example, Chithrani et al. found a saturation of particle internalization for HeLa cells after 5 h [23].

The observed correlation of the increase in internalization or cell-association of particles with the administered dose was also detected in a recent study applying ICP-MS to particle quantification [24]. Depending on the ratio between the extracellular and intracellular particle concentration, the establishment of an equilibrium state can be expected, resulting in a certain number of particles accumulating intracellularly. All in all, the results from the mentioned studies indicate that, depending on exposure time and administered dose, the number of particles per cell generally varies between 10^2^ and 10^5^, not accounting for differences in cell or particle type. The results presented here agree well with these previously published findings. Due to the influence of time and extracellular particle concentration, it is important to keep experimental conditions constant for all NP sizes when estimating intracellular NP doses, especially when internalization efficiency is correlated with particle size. For example, Chithrani et al. concluded that 50 nm particles are internalized with higher efficiency than smaller or larger particles [23]. In this case, the authors did not specify the administered particle concentration and it is unclear if they used identical mass concentrations or particle number concentrations. Assuming the former, the cells would have been exposed to increasing particle concentrations with decreasing particle size. Taking this into account, the larger particles are internalized with higher efficiency [54].

On the other hand, if the concentration value refers to particle number, a comparison between the administered particle number and the internalized particle number implies that uptake efficiency into cells is rather low. A low uptake efficiency was, for example, found by Höcherl et al., concluding that HeLa cells internalized only 10^−3^% of the initially added negatively charged 160 nm poly(methyl methacrylate) particles after 2 h based on flow cytometry and CLSM characterization [55]. In addition to the administered dose, the delivered dose is relevant when calculating uptake efficiency. Gottstein et al. quantified the uptake of 250, 400, and 1000 nm fluorescent polystyrene particles in J774 macrophages and reported that the number of internalized particles per cell was greater for the smaller particles [49]. More specifically, the delivered dose of the smaller particles was of two orders of magnitude higher than the dose of the larger particles.

In this study, by application of the computational ISDD model [22], the delivered particle dose was determined to be similar for both NP sizes, considering separated particles as well as agglomerates of up to four particles, and was approximately 20% of the administered particle number. Thus, taking the above described agglomeration models into account, 0.1% up to 5.8% and 0.3% up to 0.9% of the delivered 25 nm and 85 nm NPs entered the cells, respectively. With regard to the potential internalization process, some studies on fibroblasts indicate that clathrin-coated pits cover up 2% of the cell surface and 1% of the membrane is internalized per minute [56, 57]. When transferred to the A549 cells used in this study, it can be deduced that 1.5 × 10^4^ vesicles are internalized within 5 h, corresponding well to the number of particles observed to be internalized by the cells. The delivered particle concentration in the fluid column (*h* = 10 *μ*m) surrounding the cells was determined to be approximately 2.3 × 10^12^ NPs mL^−1^. Based on an average cell volume of 1600 *μ*m^3^, it was calculated that the cells internalized 2.5 × 10^11^–4.8 × 10^12^ particles mL^−1^. Thus, under the conditions applied, the cells did not appear to accumulate particles in excess of the delivered particle concentration.

### 4.2. Quantification of NP Uptake via Processing of STED Images

To interpret the results on particle internalization gathered by various methods, the strengths and limitations of these techniques have to be defined, in addition to considering the NP dose and uptake efficiency. First of all, in this study, like in others [55], a large difference in the number of internalized objects from cell to cell was observed. One possible explanation for this is the large variation in cell size (Additional file 2). An additional explanation for this finding, which is that the cell cycle influences uptake efficiency, was reported by Kim et al. [58].

Regarding the STED technique used in this study, high laser intensities are used that induce photobleaching of the fluorescently labeled NPs, which might lead to an underestimation of the number of internalized particles especially in the case of the smaller particles that had a lower overall fluorescence intensity than the larger particles. The number of internalized particles was likely rather underestimated during the process of quantifying internalized NPs, because only objects that were completely inside the segmented cell region of interest (ROI) were counted to ensure that the NPs attached to the cell surface were excluded. In super-resolution microscopy, data acquisition on a cell-by-cell basis, as well as image processing, is relatively time-consuming. For this reason it is not a high throughput technique like flow cytometry. Consequently, this results in lower quality statistics and limitations for comparative studies, such as uptake kinetics. An alternative approach used to quantify the internalization of particles that are smaller than the classical optical resolution limit using light microscopy was presented by Torrano et al. [59]. Their approach consisted of utilizing specially developed ImageJ macro (Particle_in_Cell-3D) and prior knowledge about the fluorescence intensity of single particles to analyze confocal image stacks of single cells, which had been exposed to 100 nm polystyrene particles. Employing this method, the fraction of internalized compared to membrane-associated particles was determined to be 92% after 5 h 45 min in HeLa cells. In order to confirm the results obtained from the quantification procedure, the application of a super-resolution microscopy technique (STED) was necessary. In the present study, the quantification of particles internalized by cells in the absence of serum was not possible because large NP agglomerates formed covering the cell membrane of cells in microscopy samples. Applying the watershed algorithm did not permit separation of these agglomerates, because the intensity distributions produced by single particles merged in the case of larger agglomerates. Thus, dividing local minima, detectable by the watershed algorithm, disappeared. Quantifying single particle events under these conditions was therefore not possible. Thus, internalization efficiencies of the NPs could not be compared. However, based on CLSM images, it appeared that more particles/particle agglomerates entered the cells under serum-free conditions. This observation is corroborated by a FACS study, in which A549 cells were found to have increased uptake levels of SNPs under serum-free conditions [11]. The authors concluded that the observed tendency of particles to adhere more strongly to the cell membrane under serum-free conditions contributes to the increase in NP uptake. It is important to note that at the particle concentration (9.2 × 10^10^ particles mL^−1^) employed in our study, which is equivalent to mass concentrations of 1.2 *μ*g SiO_2_ mL^−1^ (25 nm SNPs) and 50 *μ*g SiO_2_ mL^−1^ (85 nm SNPs), no morphological changes or membrane damage was observed that might contribute to the elevated uptake efficiency observed in CLSM images. It cannot, however, be excluded that serum-starving effects might influence cellular responses and uptake processes [60, 61].

### 4.3. Influence of Serum Proteins on NP Agglomeration and Cytotoxicity

According to SEM analysis, SNPs were found to bind to the membrane of A549 cells. The amount of particles detected on top of the cells appeared to be positively correlated with NP concentration and was much greater in the presence of serum-free medium than in the presence of serum supplemented medium although as stated above, in absence of serum no agglomeration of NPs was detected by light scattering and analytical centrifugation analyses. Our results corroborate the findings of Lesniak et al. who also observed strong adhesion of SNPs to the membrane of A549 cells in serum-free medium [11, 12]. They also detected agglomeration of SNPs in the absence of serum proteins by TEM analysis of A549 cells, although a high degree of agglomeration was not observed by light scattering analysis. The authors concluded that cell protrusions, creating some entanglement on top of the cell surface, entrapped the NPs [11].

In this study, in presence of serum no cytotoxicity or change in cell morphology could be observed even at the highest SiO_2_ concentration used. In absence of serum, membrane damage, measured as LDH release, was observed only after exposition to the 25 nm NPs at the highest SiO_2_ concentration tested (200 *μ*g mL^−1^). The results indicate that significant membrane damage can also be expected at 100 *μ*g mL^−1^. Therefore, care was taken to perform the uptake studies at subcytotoxic concentrations in order to exclude an influence of membrane damage on unspecific or passive NP uptake [11]. It has been previously reported that the presence of proteins on the surface of SNPs has a protective effect against silica-induced hemolysis and cytotoxicity [13, 62]. Also, Wang et al. have shown that the protein corona protects the cells from damage until the corona proteins are degraded within lysosomes [63]. We observed that the membrane damage induced by 25 nm SNPs in serum-free medium was reduced not only by addition of serum, like also observed by Kim et al. [64], but also after addition of single-serum proteins like BSA (bovine serum albumin) (Additional file 5). These results agree well with the research performed by Gualtieri et al. which revealed a reduced cytotoxicity of SNPs in the presence of BSA and demonstrated that the surface coating of the particles is primarily responsible for the protective effect [62]. In this study, the absolute value of the zeta potential was found to be reduced in the presence of serum, and thus it is postulated that serum components, like proteins, adsorb to the NP surface. Adsorption of proteins to the NP surface results in the electrosteric stabilization of the particle, preventing particle agglomeration, even in vicinity of the cell surface. The protein corona might also prevent the binding of counter ions, which would reduce repulsive surface charges of the particles. Light scattering analysis indicated that agglomerates formed when higher particle concentrations were dispersed in serum-containing medium (Additional file 1). This trend might explain the contrasting reports on particle agglomeration behavior. In other studies, cytotoxicity, DNA damage, and ROS production were reported after exposing cells to SNPs in serum-containing medium. In addition, lipid peroxidation and disruption of model membranes were found [14, 65]. The divergent agglomeration behavior in serum-containing medium might also be due to various synthesis protocols and stabilization of SNPs. This should be taken into account when comparing results of different studies. In comparison to other studies using commercial nanoparticles or even larger particles [11, 64], the nanoparticles used in this study were custom-made, corroborating the generality of the protective effect of the protein corona.

Our results raise the question of whether intracellular particles, accumulated at relatively low numbers, are generally able to affect the cells or if this is mediated by particles interacting with the cell surface. If one considers particle distribution, a much larger amount of particles is present close to the extracellular side of the cytoplasmic membrane than inside the cell. After exposition of cells to 9.2 × 10^10^ NPs mL^−1^ and considering particle sedimentation, 6% and 53% of the cell surface would be covered by 25 nm and 85 nm NPs, respectively. After internalization of ~7000 particles (25 nm SNPs), a volume of 0.057 *μ*m^3^ is occupied by NPs, corresponding to 3.5 × 10^−5^ part of the cell volume. Nevertheless, in this study, membrane damage was only detected at high external NP concentrations (> 50 *μ*g mL^−1^ of 25 nm SNPs SiO_2_ corresponding to 9.2 × 10^12^ NPs mL^−1^) in the absence of serum. Considering 22% particle sedimentation, at that particle concentration, the cellular surface would be more than completely covered by SNPs, indicating that membrane damage in absence of serum is initiated by direct contact of particles to the cellular surface accompanied by interaction of particles with membrane constituents.

## 5. Conclusions

This study demonstrated for the first time that quantitative estimates of the number of NPs internalized by epithelial cells can be extracted from 3D STED image stacks of entire cells via image processing. The approach used here was based on the number of intracellular fluorescent objects instead of the fluorescence intensity associated with one single cell. The internalization of 25 nm and 85 nm SNPs exposed to A549 lung epithelial cells was compared using equal particle number concentrations. Uptake studies were performed at subcytotoxic concentrations in order to exclude an influence of membrane damage on NP uptake. 10^2^-10^3^ particles per cell were determined after 5 h exposure in serum-containing medium, taking the number of particles into account that were contained within agglomerates. Although the intracellular particle concentration exceeded the initially administered particle concentration, no significant intracellular accumulation of particles above the delivered particle concentration, as determined by application of the ISDD model, was observed. By providing quantitative analyses of administered, delivered, and intracellular NPs, our study contributes to quantitative insights into nanoparticle-cell interactions. This knowledge is essential for risk assessment and safe by design approaches in nanotechnology. Future* in vitro* studies using various initial particle number concentrations and exposition times are necessary in order to elucidate uptake kinetics and relationships between intracellular and extracellular particle concentrations.

## Supplementary Material

The Supplementary Material gives further information on the concentration dependent NP agglomeration behavior in complete cell culture medium analyzed by light scattering and analytical centrifugation (Additional file 1). It also gives further information on the quantification data generated during STED analysis, by indicating the number of objects per cell and the corresponding cell data (Additional file 2), histograms of the lateral widths of internalized objects (Additional file 3), and data on the estimated number of NPs per cell after application of two and three-dimensional agglomerate models (Additional file 4). Furthermore, data on the protective effects of bovine serum albumin on the NP induced membrane damage is given (Additional file 5).

## Figures and Tables

**Table 1:** Physicochemical properties of SNPs used in the study.

Sample	Diameter EM [nm]	Hydrodynamic diameter [nm]			Zeta potential [mV]		BET surface area [m^2^g^−1^]
		Water	DMEM	Water	DMEM	DMEM + 10% FBS	
Si-25	25 ± 3	21 ± 3	28 ± 13	−24	−17 ± 4	−3 ± 2	106
Si-85	84 ± 7	71 ± 15	86 ± 16	−42	−21 ± 2	−14 ± 4	37
Si-25-FD	24 ± 2	24 ± 4	35 ± 8	−31	−5 ± 3	−8 ± 2	108
Si-85-FD	85 ± 8	79 ± 18	77 ± 15	−38	−30 ± 2	−7 ± 4	37

**Table 2:** Sedimentation and diffusion simulation results.

Particles/cluster	Deposited					
	1		3		4	
Particle diameter	Fraction (%)	Particle number (cm^−2^)	Fraction (%)	Particle number (cm^−2^)	Fraction (%)	Particle number (cm^−2^)
25 nm	26.5	1.22*E*10	19.4	8.92*E*9	18.3	8.43*E*9
85 nm	20.4	9.40*E*9	21.3	9.78*E*9	20.8	9.56*E*9

Results of the ISDD sedimentation model for SNPs with two different diameters (25 and 85 nm) in 1 mL solution with a mass concentration corresponding to 9.2 × 10^10^ particles mL^−1^. Simulation results for deposited particles are given as fraction of delivered particles (%) and particle number of deposited particles per cm^2^. The number of deposited particles displays little dependency on the number of particles per cluster for small cluster sizes and is in the same range for both particle sizes.

**Table 3:** Quantification of internalized SNP.

	Number of objects/cell	Number of objects/cell area (*μ*m^−2^)	Number of cells	Total number of objects	Mean intensity (a.u.)
Si-25-FD	117 ± 126	0.1 ± 0.1	21	2465	3.0*E*10 ± 9*E*3
Si-85-FD	338 ± 171	0.3 ± 0.2	17	5742	4.7*E*10 ± 8*E*3

Internalized SNPs were quantified by image segmentation of 3D stacks of whole A549 cells. The number of segmented objects in the NP channel is given per cell and per cell area. Also specified is the number of analyzed cells, the total number of objects found in all cells, and the mean fluorescence intensity (arbitrary units) of the objects. After exposition of cells to larger particles, a slightly higher number of objects were found inside the cells. The mean intensity values reflect the stronger fluorescence of the larger particles.
