# Supplementary material for: Quantification of Internalized Silica Nanoparticles via STED Microscopy
Source: Biomed Res Int. 2015 Jun 1;2015:961208. doi: 10.1155/2015/961208 (PMC4466362; doi:10.1155/2015/961208)
Supplement: Supplementary file 1 — The Supplementary Material gives further information on the concentration dependent NP agglomeration behavior in complete cell culture medium analyzed by light scattering and analytical centrifugation (Additional file 1). It also gives further information on the quantification data generated during STED analysis, by indicating the number of objects per cell and the corresponding cell data (Additional file 2), histograms of the lateral widths of internalized objects (Additional file 3), and data on the estimated number of NPs per cell after application of two and three-dimensional agglomerate models (Additional file 4). Furthermore, data on the protective effects of bovine serum albumin on the NP induced membrane damage is given (Additional file 5). [file 961208.f1.pdf]

Additional files

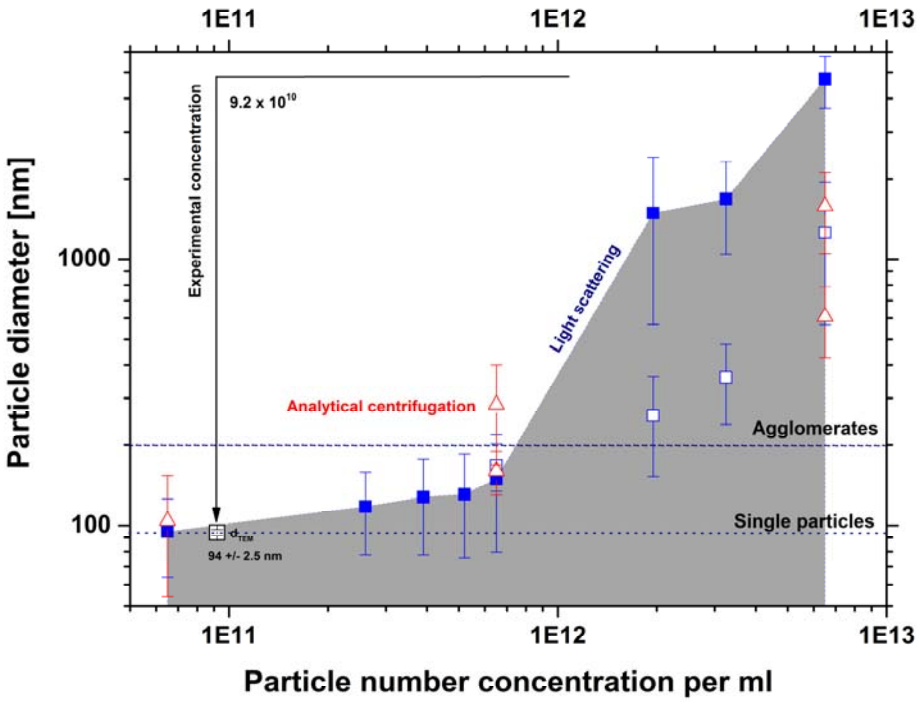

Additional file 1 – Agglomeration behavior of SiO_2 nanoparticles as a function of particle concentration in cell culture medium

Silica nanoparticles (d_{EM} = 94 ± 2.5 nm, black open squares) were incubated in DMEM + 10 % FBS for t = 5 h to investigate the agglomeration behavior as a function of particle concentration (concentration range: $6.5 \cdot 10^{10}$ - $6.5 \cdot 10^{12}$ particles per ml). Light scattering (blues squares) and analytical centrifugation (red triangles) were employed to measure and calculate the (hydrodynamic) particle diameter. Under experimental conditions (particle number concentration: $9.2 \cdot 10^{10}$), neither light scattering experiments nor analytical centrifugation indicated a significant amount of particle agglomeration. At higher particle concentrations (> $2 \cdot 10^{12}$ particles ml^{-1}), agglomeration of particles was detected, with agglomerate sizes in the range of several hundred nanometers up to > 4 µm. Error bars represent the standard deviation of the particle size.

Si-25-FD objects/cell	IsoVolume cell (μm^3)	cell area (μm^2)	Si-85-FD objects/cell	IsoVolume cell (μm^3)	cell area (μm^2)
565	5.654000E+02	888	604	1.176410E+03	1407
36	1.721330E+03	1226	471	1.680680E+03	1653
22	2.261500E+03	1089	524	1.491940E+03	1209
9	9.011600E+02	1847	230	1.116830E+03	593
225	1.751700E+03	658	238	1.735560E+03	795
124	1.848460E+03	1465	131	2.139590E+03	742
15	2.064790E+03	819	431	2.466130E+03	1137
76	1.768650E+03	591	396	1.067330E+03	1261
28	7.782300E+02	942	55	1.034590E+03	1546
147	1.306120E+03	991	386	1.857310E+03	1622
27	2.590470E+03	1883	122	9.473500E+02	807
83	6.282300E+02	834	211	1.043310E+03	1105
53	2.104180E+03	827	225	1.522490E+03	1315
55	2.104180E+03	670	645	8.414900E+02	1364
124	9.584000E+02	1077	415	1.065980E+03	1158
58	1.37E+03	697	250	2.778680E+03	1123
68	2.249340E+03	1258	408	2.982320E+03	1320
281	2.598450E+03	1335			
156	7.434600E+02	1080			
166	2.164770E+03	1336			
147	1.625110E+03	1554			

Additional file 2 – Quantification analysis data.

Values of objects per cell, cell volume and cell area as determined by image analysis (as described in the method section) are given.

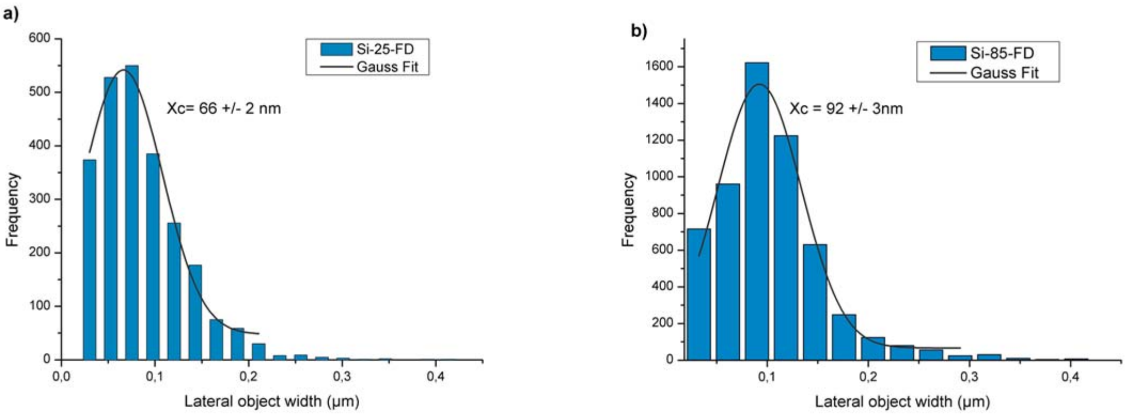

Additional file 3 – Histogram of lateral widths of internalized objects measured after exposition of A549 cells to a) Si-25-FD and b) Si-85-FD.

Histograms show the lateral widths (WiLatC) of objects, measured by application of the Huygens software (For more information see: http://www.svi.nl/ObjectAnalyzerGeometry). The peak position (x_c) of the Gaussian fit in b) at 92 nm indicates that mainly single NPs were measured.

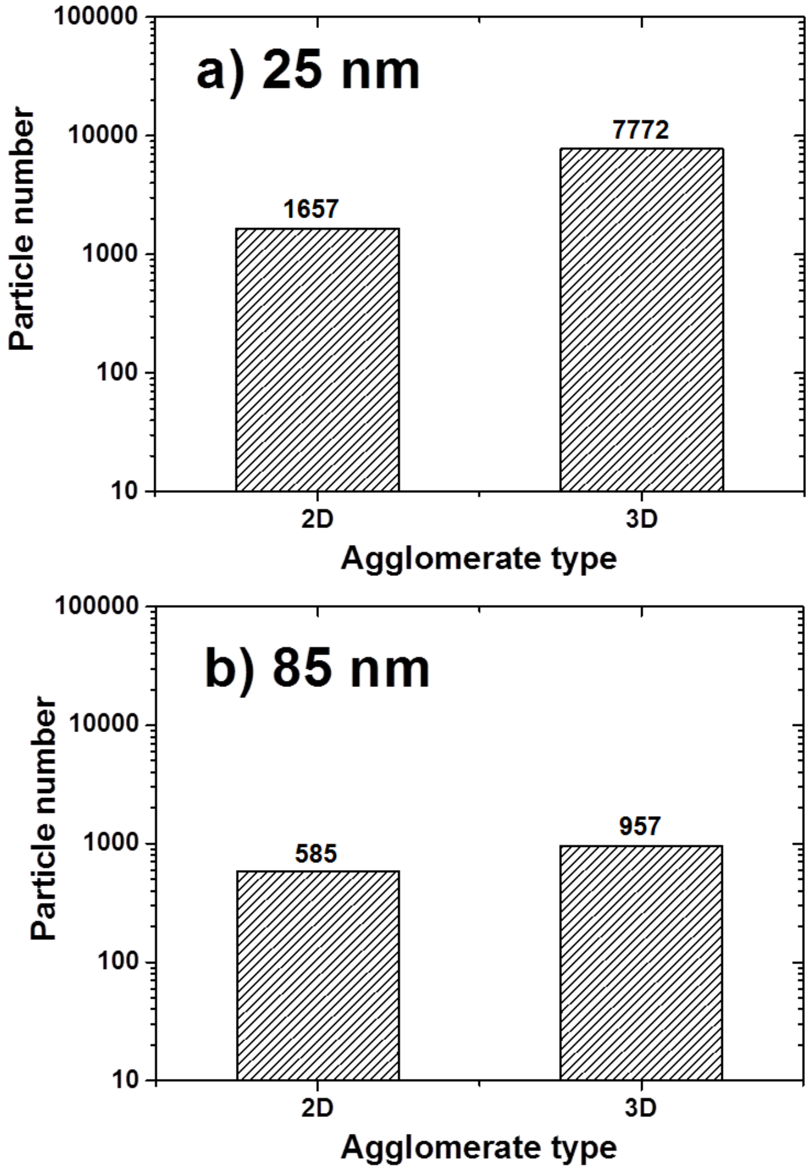

Additional file 4 – Estimated NP number per cell, according to a two-dimensional (2D) and a three-dimensional (3D) agglomerate model. a) 25 nm particles, b) 85 nm particles.

The NP number per cell was calculated assuming that the particles form either two- or three-dimensional agglomerates. Depending on the agglomeration model, the

maximal amount of nanoparticles per agglomerate was calculated under the assumption that the silica nanoparticles are hard spheres and (according to Kepplers conjecture [64]) that no greater average density than that of hexagonal or cubic close packaging can be achieved (optimal packing density: 2D ~ 90.69 %, 3D ~ 74 %). Thus, for each lateral width of internalized objects, the maximum number of particles was calculated, added up to yield the total particle number, and divided by the number of cells to yield the mean NP number per cell.

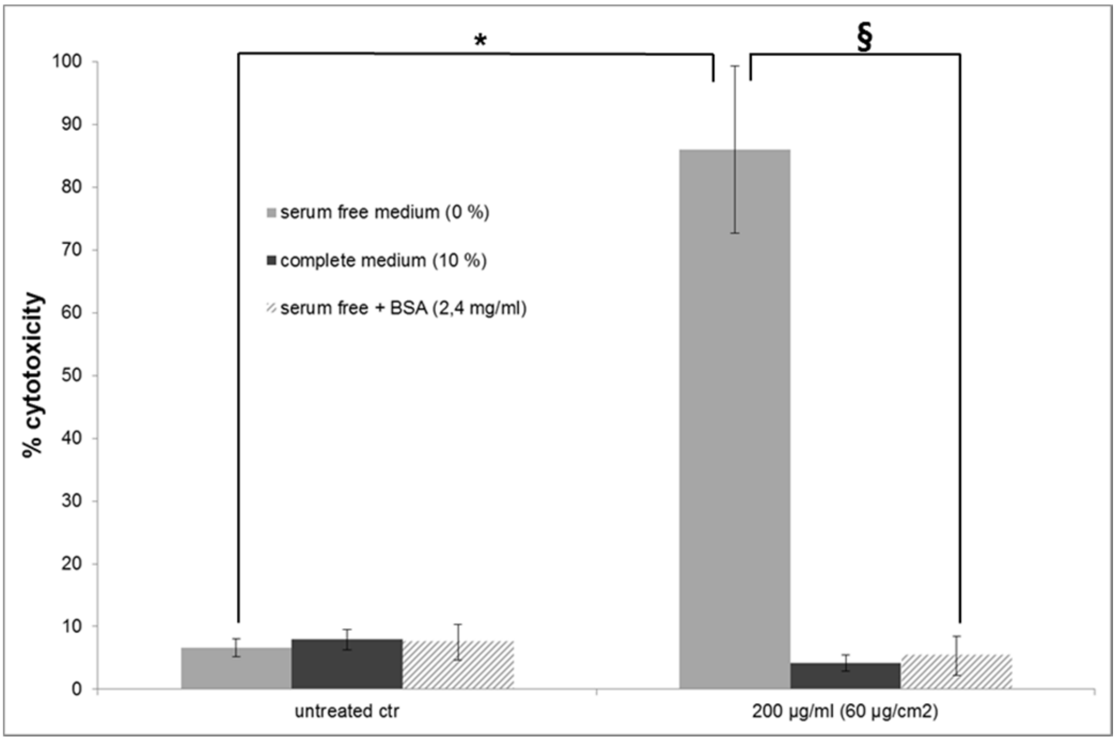

Additional file 5 – BSA prevents membrane damage induced by Si-25 in absence of serum.

A549 cells were incubated with 200 µg ml^{-1} of Si-25 particles in complete, in serum free or in serum free medium supplemented with 2.4 mg BSA ml^{-1}. LDH assay was performed after 5 h incubation, using the culture supernatant. Exposure to 200 µg ml^{-1}

Si-25 in serum free medium induced cell damage in 90 % of A549 cells compared to untreated controls (ctr). Supplementation of serum free medium with 2.4 mg BSA ml^{-1}, corresponding to the BSA concentration present in complete growth medium (10% serum), reduced the particle induced cytotoxicity to control levels. Error bars represent standard deviation of three independent experiments. *significantly different from untreated control, $p < 0.05$; §significantly different from serum free medium, $p < 0.05$.
